# A Comparative In Vitro Evaluation of the Anti-Inflammatory Effects of a *Tisochrysis lutea* Extract and Fucoxanthin

**DOI:** 10.3390/md19060334

**Published:** 2021-06-11

**Authors:** Elisabetta Bigagli, Mario D’Ambrosio, Lorenzo Cinci, Alberto Niccolai, Natascia Biondi, Liliana Rodolfi, Luana Beatriz Dos Santos Nascimiento, Mario R. Tredici, Cristina Luceri

**Affiliations:** 1 Department of NEUROFARBA, Section of Pharmacology and Toxicology, University of Florence, Viale Pieraccini 6, 50139 Florence, Italy; elisabetta.bigagli@unifi.it (E.B.); mario.dambrosio@unifi.it (M.D.); lorenzo.cinci@unifi.it (L.C.); 2 Department of Agriculture, Food, Environment and Forestry (DAGRI), University of Florence, Piazzale delle Cascine 18, 50144 Florence, Italy; alberto.niccolai@unifi.it (A.N.); natascia.biondi@unifi.it (N.B.); liliana.rodolfi@unifi.it (L.R.); lulibia.17@gmail.com (L.B.D.S.N.); mario.tredici@unifi.it (M.R.T.); 3 Fotosintetica Microbiologica S.r.l., Via di Santo Spirito, 14, 50125 Florence, Italy

**Keywords:** microalgae, *Tisochrysis lutea*, fucoxanthin, inflammation, RAW 264.7, microRNA

## Abstract

In this study, we compared the effects of a *Tisochrysis lutea* *(T. lutea)* F&M-M36 methanolic extract with those of fucoxanthin (FX) at equivalent concentration, on lipopolysaccharide (LPS)-stimulated RAW 264.7 macrophages. The *T. lutea* F&M-M36 methanolic extract contained 4.7 mg of FX and 6.22 mg of gallic acid equivalents of phenols per gram. HPLC analysis revealed the presence of simple phenolic acid derivatives. The *T. lutea* F&M-M36 extract exhibited a potent and concentration-dependent inhibitory activity against COX-2 dependent PGE2 production compared to FX alone. Compared to LPS, *T. lutea* F&M-M36 extract and FX reduced the expression of IL-6 and of Arg1 and enhanced that of IL-10 and of HO-1; *T. lutea* F&M-M36 extract also significantly abated the expression of NLRP3, enhanced mir-223 expression and reduced that of mir-146b, compared to LPS (*p* < 0.05). These findings indicate that *T. lutea* F&M-M36 methanolic extract has a peculiar anti-inflammatory activity against COX-2/PGE2 and NLRP3/mir-223 that might be attributable to the known anti-inflammatory effects of simple phenolic compounds found in the extract that may synergize with FX. Our data suggest that *T. lutea* F&M-M36 may serve as a source of anti-inflammatory compounds to be further evaluated in in vivo models of inflammation.

## 1. Introduction

*Tisochrysis lutea* (*T. lutea*) is a marine microalga belonging to Haptophyta, originally isolated from tropical seawater (Tahiti, French Polynesia), and currently used in aquaculture [1,2]. The presence of n-3 polyunsaturated fatty acids (PUFAs) such as docosahexaenoic acid (DHA) and eicosapentaenoic acid (EPA), vitamins, proteins, and xanthophylls such as fucoxanthin [3,4], makes this microalga an interesting source of compounds with anti-inflammatory and hypolipidemic activities [5,6,7]. Among the marine microalgae, *Tisochrysis* contains a high amount of the pigment fucoxanthin (FX) (1.8% w/w) [8]. In several in vitro and in vivo models, FX exerts anti-inflammatory effects by inhibiting pro-inflammatory cytokines and enzymes [9,10,11,12]. FX also attenuates alcohol-induced oxidative lesions and inflammatory responses [13]. However, *Tisochrysis* is also a source of phenolic compounds [14] which possess a high spectrum of biological activities including antioxidant, anti-aging, and anti-inflammatory effects [15,16,17,18,19,20]. Despite the anti-inflammatory and antioxidant effects of *T. lutea* that have been mostly attributed to FX, positive pharmacodynamic synergisms among various components, acting on different targets, cannot be excluded. Indeed, superior bioactivity of either the single component or the mixture was reported in studies on natural products [21].

The aim of this study was to perform a direct comparison between the anti-inflammatory activity of a methanolic extract of *T. lutea* F&M-M36 and FX at equivalent concentrations in order to explore potential interactions among the components and pharmacological mechanisms involved. Lipopolysaccharide (LPS)-stimulated RAW 264.7 mouse macrophages were used as an in vitro model of inflammation.

## 2. Results

### 2.1. Characterization of the T. lutea F&M-M36 Methanolic Extract

The amount of FX in the *T. lutea* F&M-M36 methanolic extract was 4.7 mg/g dry weight. The extract was also analyzed for the total soluble phenolic content, using gallic acid as a reference. The *T. lutea* F&M-M36 methanolic extract contained 6.22 ± 0.05 mg GAE/g dry weight.

**Figure 1:**
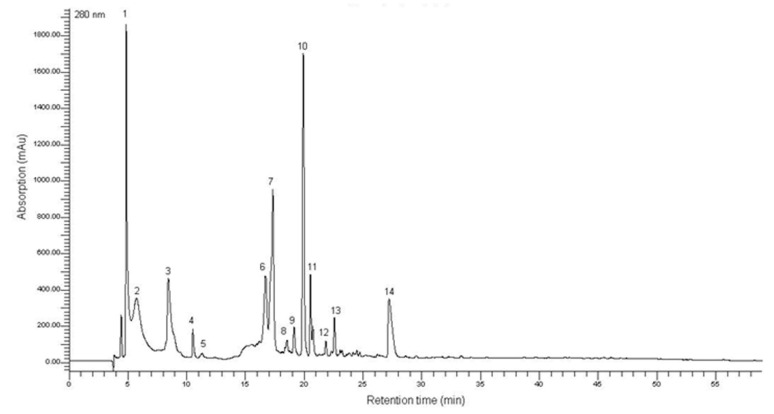
Chromatograms obtained by high-performance liquid chromatography coupled to a diode array detector—HPLC-DAD (280 nm) of methanolic extract of *T. lutea* F&M-M36. Peak 1–13: phenolic acid derivatives; pick 14: catechin derivative. The putative identification was conducted based on UV-vis absorption and retention time, in comparison with standards and literature data.

HPLC characterization showed that the *T. lutea* F&M-M36 methanolic extract contains phenolic compounds (Figure 1), with maximum absorption at 255–280 nm which eluted early in the chromatogram (retention time between 4 and 20 min, Figure 1). The spectral absorption and chromatographic behavior of these compounds are typical of simple C6 or C6-C1 phenolic skeletons, such as derivatives of hydroxybenzoic and gallic acids, as well as some aromatic amino acids. The *T. lutea* F&M-M36 methanolic extract (Figure 1) showed a little variety of phenolics, with almost all compounds showing a similar UV spectrum, compatible with the structure of simple phenolics. The putative identification was conducted based on UV-vis absorption, retention time, in comparison with standards and literature data.

### 2.2. Effects of T. lutea F&M-M36 Methanolic Extract and FX on RAW 264.7 Macrophages Viability

In order to evaluate the effects of *T. lutea* F&M-M36 methanolic extracts on cell viability, preliminary experiments were carried out using the MTS test. Unstimulated RAW 264.7 cells macrophages were exposed to different extract concentrations for 24 h. *T. lutea* F&M-M36 extract caused a significant reduction of cell viability (about 40%) when treatments were performed at 1 mg/mL, but was not toxic at concentrations in the range of 1–100 µg/mL (data not shown). On the basis of these results, 100 µg/mL was selected as the highest non-toxic concentration of *T. lutea* F&M-M36 extract for further analyses. FX was tested at concentrations equivalent to those measured in the microalgal extract at the same dilution (4.7–470 ng/mL).

### 2.3. Comparative Effects of T. lutea F&M-M36 Extract and FX on Cell Morphology

**Figure 2:**
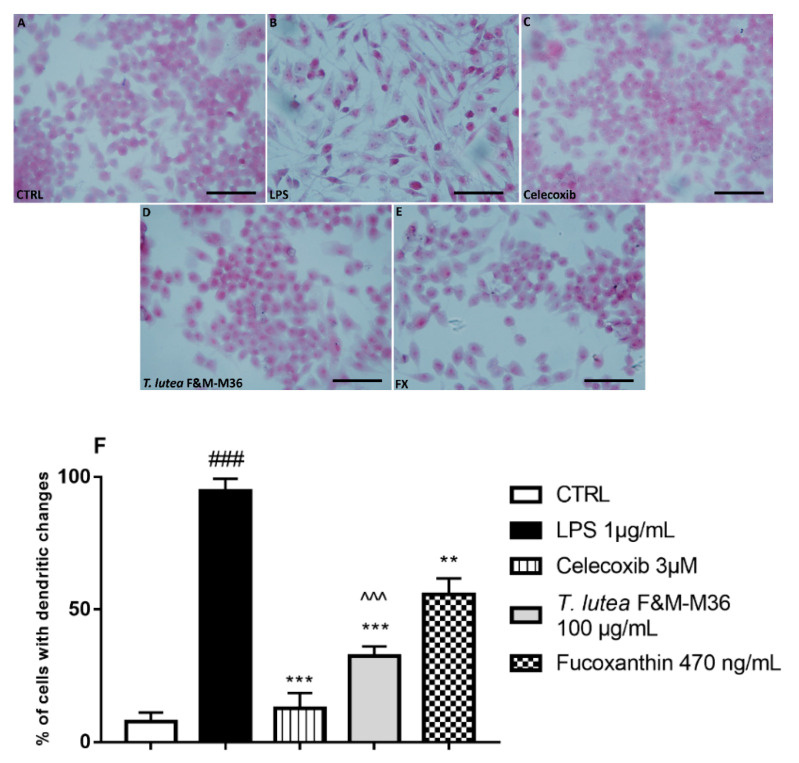
Morphology images of RAW 264.7 cells in different groups obtained by a light microscope. Hematoxylin and eosin staining of cells from different experimental groups: (**A**) Unstimulated RAW264.7 cells; (**B**) LPS-stimulated RAW264.7 cells; (**C**) LPS-stimulated RAW264.7 cells treated with Celecoxib 3 µM; (**D**) LPS-stimulated RAW264.7 cells treated with *T. lutea* F&M-M36 extract 100 µg/mL; (**E**) LPS-stimulated RAW264.7 cells treated with FX 470 ng/mL; (**F**) Percentage of cells with dendritic changes. ### *p* < 0.001 vs. unstimulated RAW 264.7 macrophages (CTRL); ** *p* < 0.01 and *** *p* < 0.001 vs. LPS ^^^ *p* < 0.001 vs. FX by ANOVA test and Dunnett’s Multiple Comparison Test. Data are expressed as mean ± SEM of five replicates. Magnification = 400×; Scale bar = 20 μm.

Hematoxylin and eosin staining showed that unstimulated RAW 264.7 cells macrophages were almost all small and round, whereas those treated with LPS were flat and spindle-shaped and showed many dendritic-like structures typical of activated macrophages [22]. The treatment with *T. lutea* F&M-M36 extract, as well as with FX, significantly reduced the number of cells with dendritic structures (*p* < 0.001 and *p* < 0.01, respectively), an effect similar to that exerted by Celecoxib (*p* < 0.001). In particular, *T. lutea* F&M-M36 extract was more effective in counteracting this effect than FX (*p* < 0.001) (Figure 2).

### 2.4. Comparative Effects of T. lutea F&M-M36 Extract and FX on PGE2 Production and COX-2 Protein Expression

**Figure 3:**
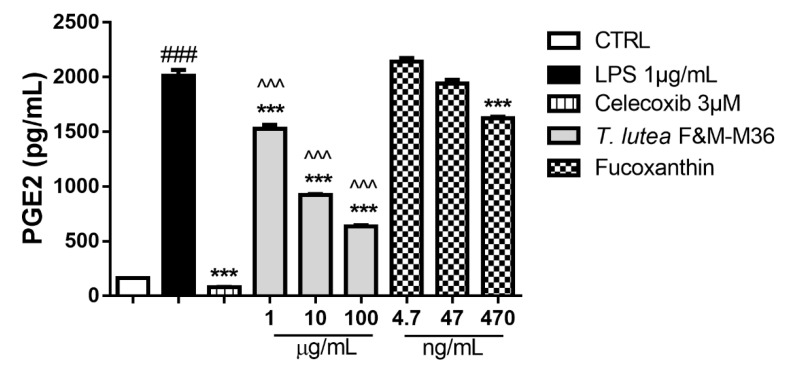
Effect of *T. lutea* F&M-M36 extract and FX on PGE2 production in RAW 264.7 stimulated with LPS for 18 h. ### *p* < 0.001 vs. unstimulated RAW 264.7 macrophages (CTRL); *** *p* < 0.001 vs. LPS ^^^ *p* < 0.001 vs. FX by ANOVA test and Dunnett’s Multiple Comparison test. Data are expressed as the mean ± SEM of four replicates.

As shown in Figure 3, the methanolic extract of *T. lutea* F&M-M36 (1–100 µg/mL) significantly decreased the LPS-induced production of PGE2 in a concentration-dependent manner, whereas FX was significantly effective (*p* < 0.05) only at the highest concentration tested (470 ng/mL). When we compared directly the PGE2 levels measured in the media from cells treated with the methanolic extract and those of the cells treated with FX at equivalent concentrations, we observed a clear and significant reduction at all concentrations (*p* < 0.001).

**Figure 4:**
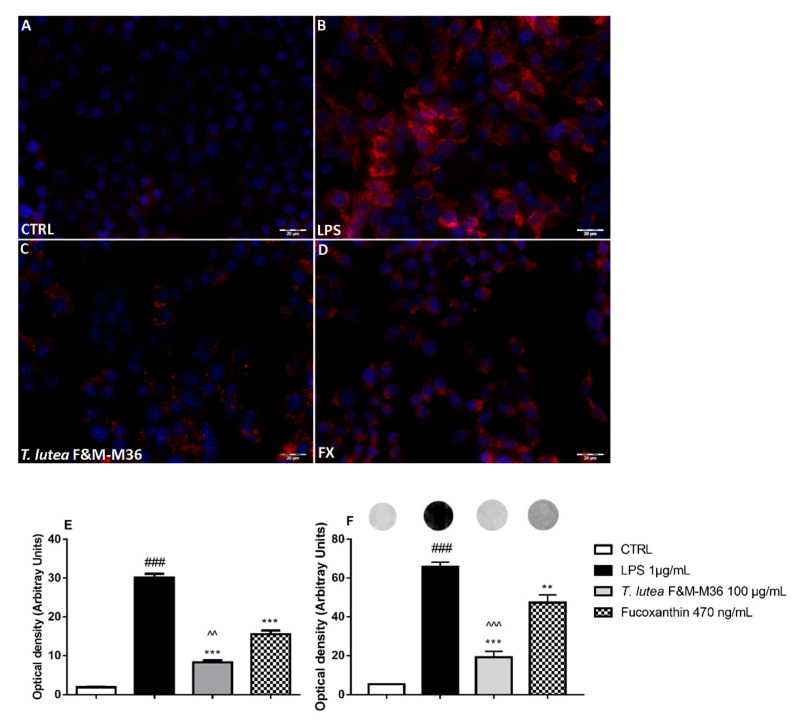
Effect of *T. lutea* F&M-M36 extract and FX on COX-2 protein expression in LPS-stimulated RAW 264.7 cells. Panels (**A**–**D**): COX-2 protein expression determined by immunocytochemistry with an anti-COX-2 antibody (red fluorescence). Nuclei were counterstained with DAPI (blue fluorescence); Magnification = 400×; Scale bar = 20 μm. Panel (**E**): Densitometric analysis of cells positive for COX-2. Panel (**F**): Densitometric analysis of dot blot results on COX-2 protein expression; above bars, representative dot blot images are shown. ### *p* < 0.001 vs. unstimulated RAW 264.7 macrophages (CTRL). ** *p* < 0.01 and *** *p* < 0.001 vs. LPS. ^^ *p* < 0.01 and ^^^ *p* < 0.001 vs. FX by ANOVA test and Dunnett’s Multiple Comparison test. Data are expressed as means ± SEM of four replicates.

Immunofluorescent staining for COX-2 protein expression (Figure 4 Panels A–D) and dot blot analyses (Panel F) demonstrated that the methanolic extract from *T. lutea* F&M-M36 significantly counteracted LPS induced COX-2 protein expression (*p* < 0.001) as it did in FX alone, although to a lower extent (*p* < 0.05) (Figure 4 panel E). Similar to the results on PGE2, *T. lutea* F&M-M36 extract also significantly decreased COX-2 protein expression compared to FX alone (*p* < 0.01).

The effects on COX-2 were much more evident at the protein level than gene level, since COX-2 mRNA expression was not significantly decreased neither by *T. lutea* F&M-M36 extract nor by FX compared to LPS-treated cells; in this regard, however, it should be highlighted that when directly compared, the mRNA expression of COX-2 was significantly decreased by *T. lutea* F&M-M36 extract compared to FX (*p* < 0.05).

### 2.5. Comparative Effects of T. lutea F&M-M36 Extract and FX on the Expression of Pro-Inflammatory and Anti-Inflammatory Genes

**Figure 5:**
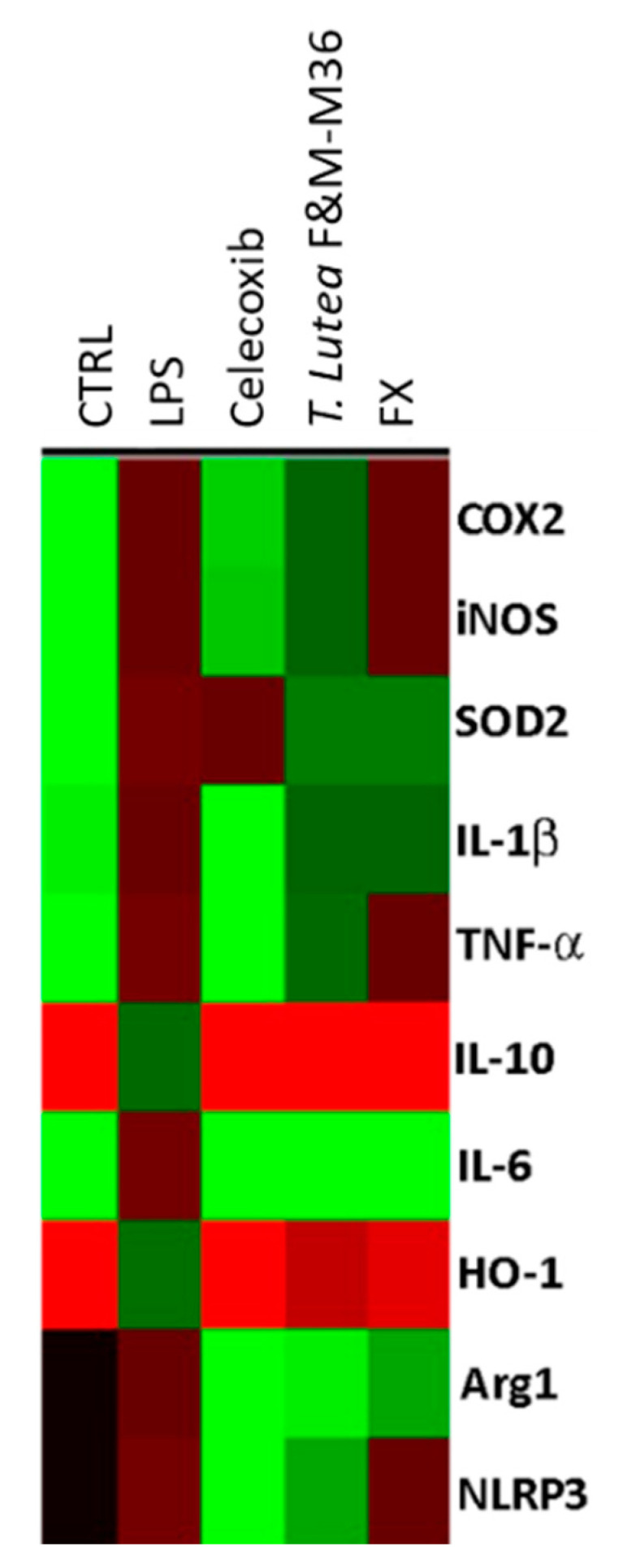
Gene expression profiles of unstimulated RAW 264.7 macrophages (CTRL), RAW 264.7 macrophages stimulated with LPS and those treated with LPS in the presence of *T. lutea* F&M-M36 extract at 100 µg/mL and FX at 470 ng/mL. Each column represents a different treatment and each row a different gene; the color code indicates down-regulation (green) or up-regulation (red) compared to LPS.

As shown in Figure 5 and Table 1, both the *T. lutea* F&M-M36 extract and FX strongly reduced the expression of IL-6 (*p* < 0.001) and enhanced that of IL-10 (*p* < 0.001) compared to LPS, and the extent of these effects were similar to those exerted by Celecoxib 3 µM. *T. lutea* F&M-M36 extract, as well as FX also reduced the mRNA expression of Arg1 compared to LPS (*p* < 0.001) and slightly enhanced that of HO-1 (*p* < 0.001); moreover, the expression of Arg1 in cells treated with *T. lutea* F&M-M36 extract was significantly lower compared to cells treated with FX (*p* < 0.001). SOD2 expression was also reduced by both *T. lutea* F&M-M36 extract and FX, compared to LPS (*p* < 0.001). *T. lutea* F&M-M36 extract and FX were ineffective in reducing the expression of iNOS, IL-1β, and TNF-α. In addition, *T. lutea* F&M-M36 extract (*p* < 0.001) but not FX significantly abated the expression of NLRP3.

### 2.6. Comparative Effects of T. lutea F&M-M36 Extract and FX on mir-146b and mir-223 Expression

**Figure 6:**
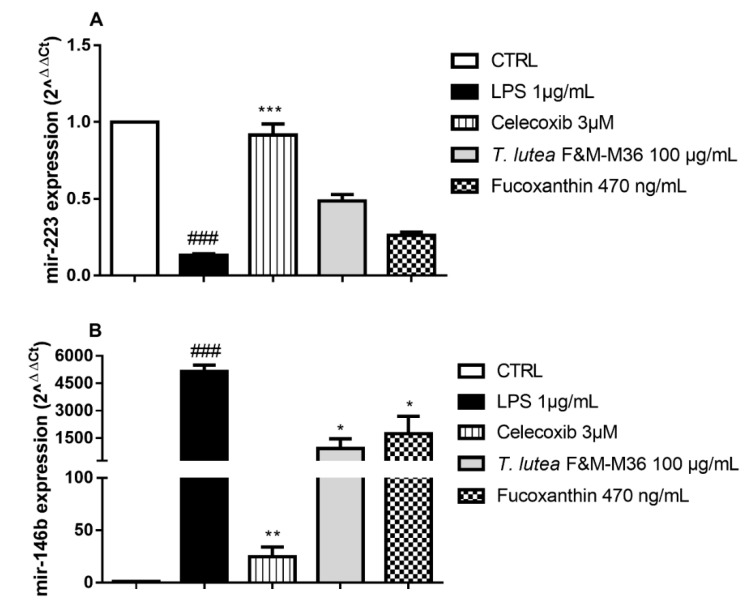
Effect of T. lutea F&M-M36 extract and FX on mir-223 (Panel **A**) and mir-146b (Panel **B**) expression in LPS-stimulated RAW 264.7 cells. ### *p* < 0.001 vs. CTRL; * *p* < 0.05, ** *p* < 0.01 and *** *p* < 0.001 vs. LPS by one-way ANOVA and Dunnett’s Multiple Comparisons test.

In RAW 264.7 macrophages stimulated with LPS, the expression of mir-146b was significantly enhanced compared to control cells (Figure 6 Panels B), whereas that of mir-223 was strongly reduced (Figure 6 Panels A); both of these effects were counteracted by Celecoxib 3 µM. *T. lutea* F&M-M36 extract, and FX showed similar effects in reducing the expression of mir-146b (*p* < 0.05). On the contrary, the expression of mir-223 was induced in cells treated with *T. lutea* F&M-M36 extract, but this difference did not reach statistical significance.

## 3. Discussion

Inflammation is a key component of several chronic human diseases such as inflammatory bowel diseases, diabetes, cardiovascular diseases, neurodegeneration, and cancer [23]. The identification of new anti-inflammatory compounds is a great challenge for the scientific community, and in this context, the microalga *T. lutea* may represent an interesting source for the discovery of novel strategies for the prevention, and even control, of inflammation.

Overall, our results demonstrate that *T. lutea* F&M-M36 methanolic extract and FX, at equivalent concentrations, exert anti-inflammatory activities by regulating a number of pro-inflammatory mediators. It is interesting to highlight that the effects on the COX-2/PGE2 axis are concentration-dependent and therefore suggestive of a pharmacological mechanism of action of *T. lutea* F&M-M36 methanolic extract and FX; the prominent reduction of COX-2/PGE2 exerted by *T. lutea* F&M-M36 methanolic extract also suggests that compounds other than FX may exert additive or synergistic effects. This is also consistent with previous reports documenting the superior activities of botanical extracts compared to single components [24]. *T. lutea* F&M-M36 methanolic extract contains polyphenols equivalent to 6.22 mg of gallic acid/g dry weight, exhibiting a much lower content of total polyphenols compared to that reported for other species, as a polyphenolic content of 515 mg GAE per 100 g DW and of 13.4 mg GAE/g EW measured in an ethanolic extract from the closely related species *I. galbana* [14,24]. These differences may be ascribed to the extraction solvents used (methanol instead of ethanol), although differences in the analyzed species and in cultivation conditions may also have contributed [25].

Despite the presence of phenolic compounds in *T. lutea* being previously described, scarce information is available on their composition; our HPLC characterization showed that *T. lutea* F&M-M36 methanolic extract contains a number of simple phenolic acids which have characteristic UV spectra (maximum absorption in the 200–290 nm range [26,27].

Simple phenolic acids derivatives of hydroxybenzoic and gallic acids have been previously proved to exert anti-inflammatory activities; gallic acid exerted inhibitory effects on LPS-stimulated PGE2 and IL-6 production and COX-2 expression in RAW 264.7 cells [27], and inhibited several NLRP3 inflammasome markers in an in vitro model of intestinal inflammation [28]. Moreover, we previously demonstrated that hydroxytyrosol, p-coumaric acid, or foods rich in simple phenols exhibited anti-inflammatory properties in in vitro and in vivo models of colon inflammation [18,20,29]. On the other hand, we cannot exclude the contribution of other, not characterized components of our methanolic extract. In particular, our *T. lutea* F&M-M36 biomass contains 4.1% of dry-weight polyunsaturated fatty acids (PUFAs) and 2.61% of total ω-3 [7] that are known to exert immunomodulatory and anti-inflammatory activities [30].

In addition, although FX is the main carotenoid found in *T. lutea*, other compounds such as diadinoxanthin, diatoxanthin, and β-carotene were found in an ethyl acetate extract from *T. lutea* containing a total amount of 132.8 mg of carotenoids/g of extract [31]. The anti-inflammatory activities of carotenoids such as β-carotene at relatively high concentrations (50–100 µM) have been reported in LPS-induced RAW264.7, showing effects on IL-1β, IL-6, and TNF-α; [32]. In the same model, other authors found significant effects of β-carotene 5 µM on IL-12, p40, and IL-1β expression [33].MiRNAs are endogenous non-coding RNA molecules that silence target mRNA by binding to the 3′UTR of mRNA [34]. Several miRNAs are regulated during the inflammatory process [35]; mir-223 is emerging as an important regulator of the innate immune system, and its deficiency enhances pro-inflammatory macrophage activation [36,37]. mir-223 targets NLRP3 result in reduced inflammation [38,39]. Our results pointed out a peculiar superior effect of the *T. lutea* F&M-M36 methanolic extract toward the NLRP3/mir223 axis. For the first time, we showed that *T. lutea* F&M-M36 methanolic extract has the ability to enhance the secretion of mir-223 by LPS-stimulated RAW 264.7, although to a lesser extent than the selective COX-2 inhibitor Celecoxib, and that this effect may be attributable to the phenolic content of the extract, considering the negligible effects of FX alone.

The activity of *T. lutea* F&M-M36 methanolic extract was prominent over that of FX on the COX-2/PGE2 pathway and NLRP3/mir-223 axis, whereas similar effects were observed when other inflammatory mediators were investigated. The ability to simultaneously target different biological inflammatory networks certainly represents an added value of both the extract and FX.

Macrophages polarization between M1 and M2 phenotypes is an important regulatory mechanism for inflammation. M1 macrophages are classically activated by LPS and sustain inflammation, whereas M2 or M2-like phenotypes are associated with the resolution of inflammation [40]. M1 macrophages express pro-inflammatory cytokines such as TNF-α, COX-2, and IL-6, while M2 macrophages express IL-10 and Arg1, thus exhibiting anti-inflammatory properties [41].

*T. lutea* F&M-M36 methanolic extract and FX promoted some morphological and molecular characteristics of the M2 anti-inflammatory phenotype in RAW macrophages, such as increased expression of IL-10 and Arg1 and decreased expression of IL-6. The extent of these effects is almost completely attributable to the FX content.

Previous findings indicate that FX (100 µg/mL) inhibited the secretion of IL-1β and TNF-α and promoted that of IL-10 and IFN-γ in Caco-2 cells stimulated with LPS [8]. In LPS-induced RAW 264.7, FX 15-60 µM (corresponding to about 10–40 µg/mL) significantly inhibited NO, TNF-α, and IL-6 production but slightly reduced PGE2 production [10] and inhibited NF-κB activation and MAPK phosphorylation at 12–50 µM [11]. In the same model, the half-maximal inhibitory concentration (IC50) for IL-6 production was 2.19 μM [12]. In a recent report, Kim et al. (2021) [42] found that the pretreatment of RAW 264.7 with FX 5 µM also significantly decreased LPS-induced expression of IL-6, IL-1β, and TNF-α by activating the NRF2/PI3K/AKT pathway. It is worth highlighting that all these studies were conducted with FX concentrations largely greater than ours (470 ng/mL). From a pharmacological point of view, the smaller is the concentration at which the molecule is active, the greater is its potential application. Recently, in a model of metabolic syndrome, a high-fat diet, supplemented with 12% (w/w) of freeze-dried *T. lutea,* significantly reduced plasma TNF-α levels and increased IL-10 in abdominal adipose tissue [43].

In addition, for the first time, we reported the ability of *T. lutea* F&M-M36 methanolic extract to reduce the secretion of mir-146b, and this effect was almost completely attributable to FX [44]. Increased levels of mir-146b are associated with inflammatory disease: in particular, mir-146b is increased in the serum of patients with inflammatory bowel disease and decreases after treatment with infliximab [45]; moreover, circulating mir-146b correlates with endoscopic disease activity in patients with inflammatory bowel disease [46].

*T. lutea* is not approved for human consumption, and its safety has been evaluated only in short-term studies in animal models [2,47,48]. However, *T. lutea* is currently used in aquaculture [1], and our data suggest that it could be added to animal feed not only for its high nutritional value, but also as an anti-inflammatory additive.

Overall, our results demonstrate that *T. lutea* F&M-M36 methanolic extract exerts promising anti-inflammatory activity, even more pronounced than that of FX alone, thus providing the background for conducting studies on its long-term safety and efficacy in inflammatory disease models.

## 4. Materials and Methods

### 4.1. Microalgal Biomass

The biomass of *T. lutea* F&M-M36 strain belonging to the Fotosintetica & Microbiologica (F&M) S.r.l. Culture Collection (Florence, Italy) was produced at Archimede Ricerche S.r.l. (Camporosso, Imperia, Italy). *T. lutea* F&M-M36 was cultivated in F medium [49] in GWP^®^-II photobioreactors [50] in a semi-batch mode. The lyophilized biomass was stored at −20 °C until extraction.

### 4.2. Microalgal Extract Preparation

An aliquot of 250 mg of lyophilized *T. lutea* F&M-M36 biomass was extracted in 30 mL of methanol, overnight, at room temperature (RT). The mixture was then sonicated twice for 3 min at the maximum power. The solvent was separated from the biomass by filtration on paper. The residual biomass was extracted again with 15 mL of methanol at 37 °C for 4 h; then, the exhausted biomass was removed by filtration on paper, and the extract (30 + 15 = 45 mL) was evaporated under vacuum. The dry residue was solubilized in DMSO to obtain a final concentration of the extract of 65 mg/mL. Fucoxanthin (purity ≥ 95%) was purchased by Sigma Aldrich (Milan, Italy).

### 4.3. Sample Preparation and HPLC-DAD Analysis for Phenols Quantification and Characterization

The extract was dried under vacuum and resuspended in 9 mL of ethanol:water solution (75:25 *v/v* adjusted at pH 2 by formic acid addition) and partitioned with 5 mL of n-hexane in order to remove chlorophylls and other pigments, which could interfere in the analysis of phenolic compounds. The procedure was repeated three times. The last partition was carried out with chloroform instead of n-hexane. The polar phase was reduced to dryness, and the residue resuspended in 0.5 mL of methanol:water solution (50:50 *v/v* adjusted at pH 2.5 by formic acid addition).

Aliquots of the samples (15 μL) were injected into the Perkin^®^ Elmer Flexar liquid chromatograph equipped with a quaternary 200Q/410 pump and an LC 200 diode array detector (DAD) (all from Perkin Elmer^®^, Bradford, CT, USA). The stationary phase was composed by a reverse-phase Agilent^®^ Zorbax^®^ SB-18 column (250 × 4.6 mm, 5 µm) (Agilent Technologies Inc., Santa Clara, CA, USA) kept at 30 °C. A gradient solvent system of solvent A (acidified water, 0.1% formic acid) and solvent B (acetonitrile, 0.1% formic acid), over a 59 min run in a flow rate of 0.6 mL/ min was applied: 0–5 min (0% B), 5–8 min (0–3% B), 8–53 min (3–40% B), 53–58 min (40% B), 58–59 min (0% B).

The chromatograms were acquired at 280 and 350 nm, the most common wavelengths for the analysis of phenolic compounds. The putative identification of the phenolics detected was carried out based on the retention time, UV spectral characteristics, and comparison with standards, as well as based on literature data. A calibration curve of gallic acid (R^2^ = 0.99) was used to quantify the compounds and the result of the total phenolic content was given in mg GAE/g dry weight. The analysis was conducted in triplicate.

### 4.4. Fucoxanthin Determination in the Methanolic Extract

FX content of *T. lutea* F&M-M36 extract was carried out by chromatographic analysis according to a modification of the method by Kim et al. [8]. FX separation was achieved with an HPLC 1050 (Hewlett Packard, Palo Alto, CA, USA) equipped with a C30 reverse-phase column (YCM Carotenoid, 4.6 mm × 250 mm, 5 μm particle size) (Waters, MA, USA), and a UV photodiode array detector 1050 (Hewlett Packard, Palo Alto, CA, USA). A gradient method with two eluents were used; eluent A: 81% Methyl Tert-Butyl Ether (MTBE), 10% methanol, and 9% deionized water, and eluent B: 93% MTBE and 7% methanol. The injection volume was 20 μL with a constant flow rate of 1 mL/min, at 25 °C temperature. The detection was performed at 450 nm. The quantification was performed by internal standard calibration. Commercial FX (Sigma-Aldrich, Milan, Italy) standard solutions (20, 40, 60, 80, 100, 120 μg/mL in methanol/MTBE 4:1), with β-apo-carotenal (50 μg/mL) and Sudan Red (90 μg/mL) were prepared. The rate between the area under the peaks of FX standard solutions and the area under the internal standard peak was plotted against FX standard solution concentrations (μg/mL) to obtain a calibration curve adopted to quantify the concentration of FX in the *T. lutea* F&M-M36 extract.

### 4.5. In Vitro Model of Inflammation and Anti-Inflammatory Assay

RAW 264.7 macrophages were purchased from the American Tissue Type Culture Collection (Manassas, VA, USA) and cultured in Dulbecco’s modified Eagle’s medium (Thermo Fisher Scientific, Milan, Italy) with 10% fetal bovine serum (FBS) (Thermo Fisher Scientific) and 100 U/mL penicillin-streptomycin (Thermo Fisher Scientific), in 5% CO_2_ at 37 °C. The cytotoxicity of the extracts was first evaluated by MTS assay as previously described [18]. FX was dissolved in DMSO and diluted in a complete cell-culture medium in order to obtain the appropriate concentrations to be tested. The final concentrations of DMSO were below 0.1%, and the control cells were exposed only to DMSO 0.1%. The cultured cells were treated with lipopolysaccharide (LPS, 1 μg/mL Sigma-Aldrich, Milan, Italy) and with *T. lutea* F&M-M36 methanolic extract (1–100 µg/mL) or FX (4.7–470 ng/mL) (Sigma-Aldrich, Milan, Italy). After incubating for 18 h at 37 °C, the cells were harvested for RNA and protein extraction, and the cell medium was collected and stored at −20 °C for PGE2 determination [18,20].

### 4.6. Morphological Analysis: Hematoxylin and Eosin (H&E) Staining

RAW 264.7 were seeded in Poly-D-lysine-coated glass dishes for 24 h then treated with LPS and *T. lutea* F&M-M36 extract, FX, or Celecoxib as described above. After 18 h, cells were fixed with 4% (w/v) paraformaldehyde for 15 min at room temperature. Next, cells were washed in H_2_O and then stained with hematoxylin for 2 min, differentiated in saturated lithium carbonate solution for 30 s, stained with eosin for 2 min, and dehydrated with ethanol series (50, 75, 96, and 100%), and finally xylene. Subsequently, glass dishes were mounted on microscope slides with a mounting medium and allowed to dry. Microscopic analysis was performed with ACT-2U software program (Nikon, Instruments Europe, Badhoevedorp, The Netherlands) connected via a camera to the microscope (Optiphot-2; Nikon). Five photomicrographs were randomly taken for each sample to evaluate cell morphology. The percentage of cells with dendritic changes (number of cells with clear morphological changes/total number of cells in the field × 100) were counted using ImageJ 1.33 image analysis software (http://rsb.info.nih.gov/ij (accessed on 22 April 2021)).

### 4.7. PGE2 Determination

PGE2 levels were measured in the RAW 264.7 cell media using an ELISA kit (Cayman Chemical, MI, USA) according to the manufacturer’s specifications, and expressed as pg/mL. Celecoxib (Sigma-Aldrich, Milan, Italy) 3 µM (1.14 µg/mL), was used as a positive control.

### 4.8. RT-PCR

Total RNA was extracted from cell lysates using the Nucleo Spin^®^ RNA kit (Macherey-Nagel, Bethlehem, PA, USA) according to the manufacturer’s instructions. For first-strand cDNA synthesis, 1 µg of total RNA from each sample was reverse-transcribed. Primers were designed based on the mouse GenBank sequences for HO-1, IL-10, IL-6, IL1-β, COX-2, iNOS, TNF-α, SOD2, NLRP3, and Arg1, and are reported in Table 2. Ribosomal protein large P1 (RPLP-1) was co-amplified as the reference [18]. For each target gene, the relative amount of mRNA in the samples was calculated as the ratio to RPLP-1 mRNA [19].

### 4.9. Real-Time PCR for mir-146b and mir-223 Expression Analysis

For miRNA expression analysis, the total RNA was extracted from cell culture media by using TRIzol (Invitrogen, Carlsbad, CA, USA). Reverse-transcription of RNA was performed using the miRCURY LNA RT Kit according to the manufacturer’s instructions (Qiagen). qRT-PCR assays were carried out in a Rotor-Gene^®^Q PCR System (Qiagen) using a miRCURY LNA SYBR^®^ Green PCR Kit and miRCURY LNA miRNA PCR Assay according to the manufacturer’s instructions (Qiagen). Briefly, each reaction was performed in a final volume of 10 μL containing two μL of the cDNA, a master mix containing 5 μL of 2× miRCURY SYBR Green PCR Master Mix, 1 μL of miRCURY LNA miRNA PCR Assay, and RNase-free water. The amplification profile was: PCR initial heat activation at 95 °C for 2 min, followed by 40 cycles of denaturation at 95 °C for 10 s and combined annealing/extension at 56 °C for 60 s. The expression of mir-146b and mir-223 was normalized to RNU6B and calculated as 2-^ΔΔCt^.

### 4.10. Dot-Blotting for COX-2 Protein Expression

Cells were lysed in a 300 µL radioimmunoprecipitation assay buffer (RIPA) (Sigma-Aldrich, Milan, Italy). Total protein content was measured by using the Bio-Rad DC protein assay kit (Bio-Rad, Milan, Italy). Equal aliquots (30 μg) of proteins were applied to a nitrocellulose membrane (Millipore, Burlington, VT, USA) and allowed to dry for 30 min at RT. After blocking with 6% nonfat dry milk for 1 h at RT, the membranes were incubated overnight at RT with the Rabbit anti-COX-2 polyclonal antibody (1:200) (Cayman Chemical, MI, USA, catalog number 160126) followed by incubation with anti-rabbit IgG horseradish peroxidase-linked antibody (Cell Signaling, Danvers, MA, USA), 1:4000 for 1 h at RT. Chemiluminescence was developed by using the Immobilon Horseradish Peroxidase Substrate (Merck Millipore, Darmstadt, Germany), and immunoreactive spots were quantified using Quantity-One software (Bio-Rad Laboratories S.r.l., Milan, Italy).

### 4.11. Immunocytochemistry for COX-2 Protein Expression

RAW 264.7 cells were grown in Poly-D-lysine-coated glass dishes for 24 h then treated with LPS and compounds and extracts tested as described above. After treatment, cells were fixed with cold 4% (*w/v*) paraformaldehyde for 20 min, washed in PBS, and then incubated for 15 min with 0.1% (*w/v*) TritonX-100 and 3% Bovine Serum Albumin (BSA). Thereafter, the cells were incubated with Rabbit anti-COX-2 polyclonal antibody (1:200) (Cayman, Ann Arbor, MI, USA, catalog number 160126) at 4 °C overnight, followed by the fluorescent secondary antibody: AlexaFluor 586 goat anti-rabbit (1:333) (Invitrogen, Carlsbad, CA, USA). Nuclei were also counterstained with DAPI dye (Sigma-Aldrich, Milan, Italy). Microscopic analysis was performed with an Olympus BX63 microscope equipped with a Metal Halide Lamp (Prior Scientific Instruments Ltd., Cambridge, UK) and a digital camera, Olympus XM 10 (Olympus, Milan, Italy).

### 4.12. Statistical Analysis

Data were analyzed by ANOVA test and Dunnett’s Multiple Comparison test and expressed as the means ± standard error (SEM) of four independent experiments. All analyses were carried out using GraphPad Prism 7.0 (GraphPad Software, San Diego, CA, USA). *p* values less than 0.05 were considered significant.

## Figures and Tables

**Table 1:** Effect of *T. lutea* F&M-M36 extract at 100 µg/mL and FX at 470 ng/mL on COX-2, iNOS, SOD2, IL-1β, TNF-α, IL-10, IL-6, HO-1, Arg1, and NLRP3 mRNA expression in LPS-stimulated RAW 264.7 cells.

Gene	CTRL	LPS	Celecoxib	*T. lutea* F&M-M36 Extract	FX
COX-2	0.09 ± 0.01	0.86 ± 0.03 ^###^	0.37 ± 0.03 ***	0.81 ± 0.01 ^^^	0.91 ± 0.01
iNOS	0.02 ± 0.01	1.25 ± 0.03 ^###^	0.51 ± 0.02 ***	1.10 ± 0.06	1.20 ± 0.00
SOD2	0.04 ± 0.02	0.64 ± 0.00 ^###^	0.62 ± 0.01	0.44 ± 0.02 ***	0.44 ± 0.01 ***
IL-1b	0.36 ± 0.00	0.97 ± 0.02 ^###^	0.31 ± 0.01 ***	0.90 ± 0.01	0.92 ± 0.01
TNF-a	0.27 ± 0.03	0.76 ± 0.03 ^###^	0.20 ± 0.02 ***	0.69 ± 0.02	0.82 ± 0.01
IL-10	0.62 ± 0.03	0.16 ± 0.00 ^###^	0.71 ± 0.03 ***	0.61 ± 0.06 ***	0.62 ± 0.03 ***
IL-6	0.02 ± 0.01	0.74 ± 0.03 ^###^	0.06 ± 0.01 ***	0.04 ± 0.00 ***	0.07 ± 0.01 ***
HO-1	1.04 ± 0.00	0.25 ± 0.00 ^###^	0.95 ± 0.03 ***	0.38 ± 0.01 ***	0.46 ± 0.03 ***
Arg1	0.00 ± 0.00	1.71 ± 0.01 ^###^	0.06 ± 0.00 ***	0.61 ± 0.02 ***^, ^^^^	0.91 ± 0.02 ***
NLRP3	0.00 ± 0.00	0.74 ± 0.02 ^###^	0.23 ± 0.02 ***	0.37 ± 0.03 ***^, ^^^^	0.71 ± 0.05

Data are expressed as means ± SEM of four replicates; for each target gene, the relative amount of mRNA was calculated as the ratio to RPLP-1 mRNA [19]; ^###^ *p* < 0.001 vs. CTRL; *** *p* < 0.001 vs. LPS; ^ *p* < 0.05 and ^^^ *p* < 0.001 vs. FX by one-way ANOVA and Dunnett’s multiple comparisons test.

**Table 2:** Primer sequences.

Gene	Primer Forward	Primer Reverse	Base Pair
RPLP-1	ATCTACTCCGCCCTCATCCT	CAGATGAGGCTCCCAATGTT	155
COX-2	TCCTCCTGGAACATGGACTC	CCCCAAAGATAGCATCTGGA	321
iNOS	CCCCAAAGATAGCATCTGGA	CCCCAAAGATAGCATCTGGA	305
SOD2	ACCCAAAGGAGAGTTGCTGGA	ATGTGGCCGTGAGTGAGGTT	354
HO-1	GGCTGCCCTGGAGCAGGACGT	AGGTCACCCAGGTAGCGG	165
TNFα	TAGCCCACGTCGTAGCAAAC	ACCCTGAGCCATAATCCCCT	566
NLRP3	TGGGTTCTGGTCAGACACGAG	GGGGCTTAGGTCCACACAGAA	176
ARG1	CATTGGCTTGCGAGACGTAG	CGGCCTTTTCTTCCTTCCCAG	151
IL-1β	CAGGCAGGCAGTATCACTCA	AGGCCACAGGTATTTTGTCG	350
IL-10	AGGCGCTGTCATCGATTTCTC	AGGAAGAACCCCTCCCATCA	489
IL-6	TCCTCTCTGCAAGAGACTTCC	TCCTCTCTGCAAGAGACTTCC	513

## Data Availability

The data that support the findings of this study are available on request from the corresponding author C.L. (cristina.luceri@unifi.it).
